# Renin-angiotensin system inhibitors and risk of fractures: a prospective cohort study and meta-analysis of published observational cohort studies

**DOI:** 10.1007/s10654-017-0285-4

**Published:** 2017-07-27

**Authors:** Setor K. Kunutsor, Ashley W. Blom, Michael R. Whitehouse, Patrick G. Kehoe, Jari A. Laukkanen

**Affiliations:** 1 Musculoskeletal Research Unit, School of Clinical Sciences, University of Bristol, Learning and Research Building (Level 1), Southmead Hospital, Southmead Road, Bristol, BS10 5NB UK; 2 Dementia Research Group, School of Clinical Sciences, Faculty of Health Sciences, University of Bristol, Learning and Research Building (Level 1), Southmead Hospital, Southmead Road, Bristol, UK; 3 Institute of Public Health and Clinical Nutrition, University of Eastern Finland, Kuopio, Finland; 4 Internal Medicine, Central Finland Central Hospital, Jyväskylä, Finland

**Keywords:** Renin-angiotensin system, Angiotensin converting enzyme, Angiotensin receptor blocker, Cohort study, Fracture

## Abstract

The renin-angiotensin system (RAS) represents an important target of antihypertensive medications. Angiotensin-converting enzyme inhibitors (ACEI) and angiotensin II receptor blockers (ARB), which are widely-used RAS inhibiting drugs, have been suggested to have beneficial effects on bone tissue. We aimed to assess the associations of use of ACEIs and/or ARBs with the risk of fractures using a population-based prospective cohort and a meta-analysis of published prospective cohort studies. Information on antihypertensive medication use (including both ACEIs and ARBs) were assessed in 1743 men and women of the Kuopio Ischemic Heart Disease prospective cohort study. Hazard ratios (HRs) [95% confidence intervals (CI)] of ACEIs or ARBs use with incident fractures were calculated. A total of 203 composite (hip, humeral, and wrist) fractures occurred during a median follow-up of 14.8 years. In multivariate adjusted analysis, the HR for composite fractures comparing users of ACEIs or ARBs with non-users was 1.00 (0.59–1.69). The corresponding adjusted HR for hip fractures comparing users versus non-users of ACEIs or ARBs was 0.89 (0.32–2.47). Including the current study, a total of 11 observational cohort studies involving 3526,319 participants and >323,355 fractures were included in a meta-analysis. Comparing ACEI users with non-users and ARB users with non-users, the HRs for composite fractures were 1.09 (0.89–1.33) and 0.87 (0.76–1.01) respectively. The corresponding HRs for hip fractures were 0.91 (0.86–0.95) and 0.80 (0.75–0.85) respectively. Use of RAS inhibitors was not associated with long-term risk of composite fractures in both primary and pooled analyses. Pooled evidence however suggests a beneficial effect of RAS blockers on hip fracture risk.

**Electronic supplementary material:**

The online version of this article (doi:10.1007/s10654-017-0285-4) contains supplementary material, which is available to authorized users.

## Introduction

Aging of the population is associated with an increase in age-related chronic conditions such as fractures (particularly osteoporotic fractures). These are one of the most common causes of disability worldwide and associated with high health care costs [1, 2]. Complications of fracture include morbidity, pain, limited function, reduction in health-related quality of life, as well as mortality [3]. Mortality rates in the first year following hip fracture have been reported to range from 10 to 50% [4, 5]. The prevention of fractures is therefore of public health importance.

The majority of older people with osteoporosis have co-morbidities such as hypertension and cardiovascular disease. Two major risk factors for osteoporotic fractures are reduced bone mass and falls, and these have a close relationship with hypertension [6]. Elevated blood pressure or diagnosed hypertension has been shown to be closely associated with osteoporosis, decreased bone mineral density (BMD), falls, as well as fractures [6–10]. Epidemiological evidence and studies in animal models suggest that high blood pressure is associated with vitamin D deficiency and abnormalities in calcium metabolism [11, 12], which are known to be involved in the pathophysiology of osteoporosis, falls, and fractures [13]. It therefore appears that medications that lower blood pressure may have a beneficial effect on bone tissue. Indeed, blood pressure lowering medications such as thiazides and β-blockers have consistently been shown to be associated with the reduced risk of fractures [14–17]. Furthermore, the renin-angiotensin system (RAS), that plays a vital role in regulating blood pressure and electrolyte balance [18], and the activation of which is an important contributor to systemic hypertension [19], also has effects on bone tissue. This is via the detrimental effects of angiotensin II, a primary mediator of numerous RAS functions, on the bone [20]. Studies have shown that RAS activation induces osteoporosis as well as reduces blood ionized calcium levels [20, 21]. The RAS inhibiting drugs—angiotensin-converting enzyme inhibitors (ACEI) and angiotensin II receptor blockers (ARB)—which respectively inhibit the formation and signalling of angiotensin II peptide, may have beneficial effects on bone tissue. Though improved BMD as well as reductions in fracture risk have been reported with the use of RAS inhibitors [14, 22–24], the evidence has been inconsistent. Some studies, including a previous meta-analysis, have also reported increases in fracture risk as well as bone loss [25–29], whereas others have shown no effects of RAS inhibitors on fracture risk [14, 29]. In addition, the majority of studies on the topic have been based on case–control designs [14, 24, 27], therefore the temporal relationship between the use of (i.e. exposure to) RAS inhibitors and their effect on future risk of fractures is uncertain. RAS inhibitors in addition to thiazides and β-blockers, are well established and widely used drugs for the management of hypertension in people, who are also prone to fractures; therefore, it will be clinically useful if they are proven to reduce fracture risk. In this context, this study aimed to investigate the prospective effect of RAS inhibitors (ACEIs and ARBs) on the risk of fractures using a population-based prospective cohort of 1743 middle-aged to elderly men and women from eastern Finland. Furthermore, with the availability of a number of published observational cohort studies that have evaluated the associations between RAS inhibitors and risk of fractures, this offered the opportunity to put the findings into context by performing a systematic review and meta-analysis.

## Methods

We conducted the primary cohort analyses according to STROBE (STrengthening the Reporting of OBservational studies in Epidemiology) guidelines for reporting observational studies in epidemiology (Appendix 1 of Electronic Supplementary Material) [30].

### Study population

The study population formed part of the ongoing Kuopio Ischaemic Heart Disease (KIHD) population-based prospective cohort study, which was set up primarily to investigate established and emerging risk factors for cardiovascular disease and other additional health outcomes in eastern Finland [31]. Participants comprised a randomly selected sample of 2358 participants (1007 men and 1351 women) aged 53–74 years who resided in the town of Kuo-pio or its surrounding rural communities and had baseline assessments carried out between March 1998 and December 2001. Of the 2072 potentially eligible participants, 193 refused to participate, 66 did not respond to the invitation and 39 declined to give informed consent; leaving 1774 participants for the KIHD cohort. The current analysis included 1743 participants (913 women and 830 men) with non-missing information on use of ACEIs or ARBs, relevant covariates, and fracture outcomes (Appendix 2 of Electronic Supplementary Material). The study protocol was approved by the Research Ethics Committee of the University of Eastern Finland and each participant gave written informed consent according to the Declaration of Helsinki.

### Exposure

Antihypertensive medications were classified based on antihypertensive medication classes; all antihypertensives, ACEIs or ARBs, β-blockers, calcium channel blockers, and diuretics. Data on diagnosis of chronic diseases including hypertension and the use of antihypertensive drugs were assessed by self-administered questionnaires. These were then cross-checked by a physician.

### Fracture outcomes

We included all incident fractures, representing all hip, humeral, and wrist fractures, that occurred from study entry to 2014. The endpoints assessed were incident composite, hip, and wrist fractures. Composite fractures were defined as hip, humeral, and wrist fractures. In the KIHD study, participants are under annual continuous surveillance for the development of new outcome events, including fractures [32]. No losses to follow-up have so far been recorded. Fracture incidence data were collected from the National Hospital Discharge Register data by computer linkage using Finnish personal identification codes as well as a comprehensive review of hospital records, discharge notes and diagnoses, and inpatient physician claims. The events were coded according to the International Classification of Diseases Tenth Revision (ICD-10) diagnostic codes for fractures by site.

### Assessment of risk markers

All baseline characteristics as well as risk markers were assessed during the same visit at study entry. Methods for collection of blood specimens and the measurement of lipids and biochemical analytes have been previously described in detail [33]. Briefly, besides fasting overnight before blood collection, participants were told to abstain from drinking alcohol for at least 3 days and from smoking for at least 12 h before assessment. The cholesterol content of lipoprotein fractions was measured from fresh samples after combined ultracentrifugation and precipitation, and serum triglycerides were assessed enzymatically (Boehringer Mannheim, Mannheim, Germany). Resting blood pressure was measured between 8 and 10 a.m. with a random-zero sphygmomanometer. Participants completed self-administered health and lifestyle questionnaires for the assessment of age, smoking, alcohol consumption, socio-economic status (SES), prevalent diseases, medical history, and use of medications [33]. Energy expenditure of physical activity was assessed using the validated KIHD 12-month leisure-time physical activity questionnaire [34, 35].

### Statistical analyses

#### Prospective cohort analyses

Baseline characteristics were presented as means (SD) or median (interquartile range) for continuous variables and percentages for categorical variables. Cox proportional hazard regression models were used to conduct time-to-event analyses after confirmation of the proportional hazards assumptions [36]. Antihypertensive medication use was categorised as no antihypertensive medication use, diuretics use, β-blockers use, and ACEI or ARB use with the use of dummy variables. Hazard ratios were progressively adjusted for (i) age and sex; (ii) body mass index (BMI), smoking, history of diabetes, systolic blood pressure (SBP), prevalent hypertension, prevalent coronary heart disease (CHD), history of heart failure, alcohol consumption, and use of statins or calcium channel blockers; and (iii) SES and physical activity. We evaluated effect modification by pre-specified clinically relevant characteristics using tests of interaction.

#### Systematic review and meta-analysis

We conducted a systematic review and meta-analysis of observational cohort studies using a predefined protocol and which was reported in accordance with PRISMA and MOOSE guidelines [37, 38] (Appendices 3 and 4 of Electronic Supplementary Material). Published observational population-based cohort (prospective, case cohort, nested case–control, or retrospective) studies that evaluated the associations between exposure to ACEIs or ARBs and the risk of fractures, were sought using computer-based databases (MEDLINE, EMBASE, and Web of Science) from inception to April 2017. The computer-based searches combined free and MeSH search terms and combined key words related to the exposure (e.g., “angiotensin-converting enzyme inhibitors”, “angiotensin II receptor blockers”, “anti-hypertensive drugs”) and outcome (e.g., “fracture”). There were no restrictions on language. Details of the search strategy are reported in Appendix 5 of Electronic Supplementary Material. After an initial screen of abstracts and titles by one reviewer (S.K.K.), potentially relevant articles were acquired. Each article was assessed by two independent reviewers (S.K.K., M.R.W.) using the inclusion criteria and any discrepancies regarding eligibility of an article was discussed, and consensus reached with a third author (J.A.L.). One author (S.K.K.) independently extracted data and performed quality assessments using the nine-star Newcastle–Ottawa Scale (NOS) [39] as described previously [40]. Information was extracted on study characteristics such as study design, publication year, geographical location, baseline age, duration of follow-up, sample size and number of recorded fractures, and risk estimates for the most adjusted models. A second reviewer checked data with that in original articles. Summary measures were presented as relative risks (RRs) with 95% confidence intervals (CIs). Following Cornfield’s rare disease assumption [41], hazard ratios and odds ratios were assumed to approximate the same measure of RR. Summary RRs were pooled using a random effects model to minimize the effect of between-study heterogeneity [42]. Heterogeneity was assessed using the Cochrane *χ* ^*2*^ statistic and the *I* ^*2*^ statistic [43]. A narrative synthesis was performed for studies that could not be pooled. All statistical analyses were conducted using Stata version 14 (Stata Corp, College Station, Texas).

## Results

### Baseline characteristics

Table 1 provides a summary of baseline characteristics of overall study participants and according to the development of fractures. Of 1743 study participants, 736 (42.2%) were on regular antihypertensive medication and of these, 249 (14.3%) were on ACEIs or ARBs. There were 830 (47.6%) male participants. The mean (SD) age and BMI of study participants were 63 [7] years and 27.9 (4.5) kg/m^2^ respectively. Except for age, sex, history of CHD, waist-to-hip ratio, and diastolic blood pressure, there were no significant differences in baseline characteristics between those who developed and did not develop fractures during follow-up. Participants who experienced a fracture were more likely to be older and have a history of CHD at baseline compared with those who did not experience a fracture. Males were less likely to experience a fracture compared with females.

**Table 1:** Baseline participant characteristics overall and according to the development of fractures

	Overall (N = 1743) Mean (SD), median (IQR), or n (%)	Without fracture (N = 1540) Mean (SD), median (IQR), or n (%)	With fracture (N = 203) Mean (SD), median (IQR), or n (%)	*P* value*
*Questionnaire/prevalent conditions*				
Age at survey (years)	62.9 (6.5)	62.5 (6.4)	65.2 (6.4)	<0.0001
Males	830 (47.6)	763 (50.0)	67 (33.0)	<0.001
Alcohol consumption (g/week)	48.2 (100.4)	48.5 (101.6)	46.2 (91.2)	0.755
Socioeconomic status	10.9 (4.7)	10.8 (4.7)	11.4 (4.6)	0.081
History of diabetes	140 (8.0)	123 (8.0)	17 (8.4)	0.849
Smoking status	228 (13.1)	203 (13.2)	25 (12.3)	0.731
History of hypertension	722 (41.4)	637 (41.4)	85 (41.9)	0.890
History of CHD	488 (28.0)	418 (27.1)	70 (34.5)	0.029
History of heart failure	129 (7.4)	108 (7.0)	21 (10.3)	0.088
*Use of medication*				
Antihypertensives	736 (42.2)	643 (41.8)	93 (45.8)	0.271
Beta-blockers	457 (26.2)	396 (25.7)	61 (30.1)	0.187
CCBs	210 (12.1)	182 (11.8)	28 (13.8)	0.417
Diuretics	172 (9.9)	146 (9.5)	26 (12.8)	0.135
Statins	78 (4.5)	74 (4.8)	4 (2.0)	0.066
ACEIs and/or ARBs	249 (14.3)	222 (14.4)	27 (13.3)	0.670
*Physical measurements*				
BMI (kg/m^2^)	27.9 (4.5)	27.8 (4.4)	28.1 (4.7)	0.445
WHR	0.91 (0.09)	0.91 (0.09)	0.89 (0.09)	0.034
SBP (mmHg)	135.9 (17.3)	135.8 (17.3)	136.3 (17.0)	0.694
DBP (mmHg)	81.1 (9.0)	81.4 (9.0)	79.4 (8.3)	0.004
Physical activity (kj/day)	477.6 (402.1)	482.5 (408.7)	441.0 (346.8)	0.166
*Blood biomarkers*				
Total cholesterol (mmol/l)	5.48 (0.96)	5.48 (0.95)	5.46 (1.05)	0.735
HDL-C (mmol/l)	1.25 (0.31)	1.25 (0.31)	1.28 (0.33)	0.192
Triglycerides (mmol/l)**	1.12 (0.83–1.54)	1.12 (0.82–1.54)	1.13 (0.83–1.55)	0.554
Fasting plasma glucose (mmol/l)	5.08 (1.21)	5.07 (1.18)	5.11 (1.39)	0.667

*ACEI* angiotensin-converting enzyme inhibitor, *ARB* angiotensin II receptor blocker, *BMI* body mass index, *CCB* calcium channel blocker, *CHD* coronary heart disease, *DBP* diastolic blood pressure, *GFR* glomerular filtration rate, *HDL-C* high-density lipoprotein cholesterol, *IQR* interquartile range, *SD* standard deviation, *SBP* systolic blood pressure, *WHR* waist-to-hip ratio; *, based on t-tests; **, values were log-transformed before conducting t-tests

### RAS inhibitors and risk of fractures

#### Prospective cohort analysis

**Fig. 1:**
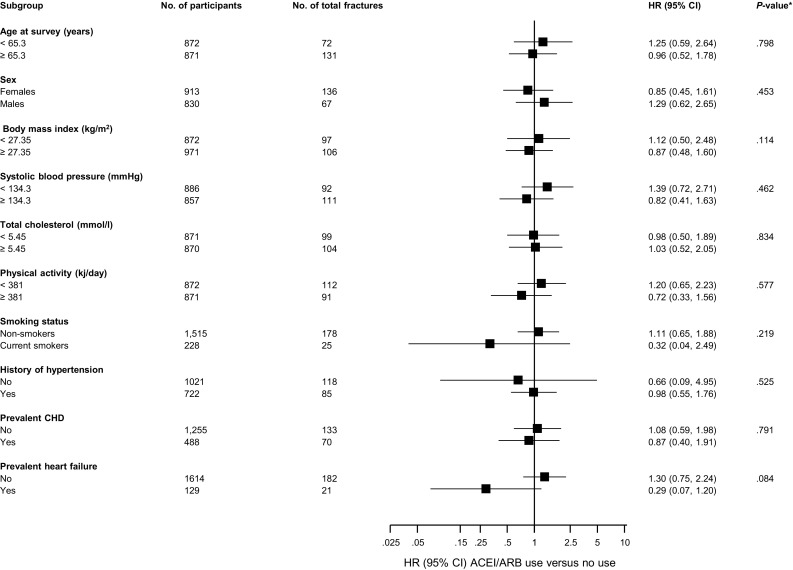
Hazard ratios for composite fractures risk comparing ACEIs or ARBs use with no use, by several participant level characteristics. Hazard ratios were adjusted for age, sex, BMI, smoking, history of diabetes, systolic blood pressure, prevalent hypertension, prevalent CHD, prevalent heart failure, alcohol consumption, and use of statins, or calcium channel blockers; *ACEI* angiotensin-converting enzyme inhibitor, *ARB* angiotensin II receptor blocker, *CHD* coronary heart disease, *CI* confidence interval, *HR* hazard ratio, *, *P* value for interaction; cut-offs used for age, body mass index, systolic blood pressure, total cholesterol, and physical activity are median values

During a median (interquartile range) follow-up of 14.8 (12.8–15.8) years, 203 incident composite fractures (annual rate 8.76/1000 person-years at risk; 95% CI 7.63–10.05) were recorded. Of the total number of incident fractures, 70 and 42 were hip and wrist fractures respectively. Comparing ACEIs or ARBs users with non-users, the age and sex adjusted HR for composite fractures was 1.00 (95% CI 0.66–1.52; *P* = 0.992), which remained non-significant following further adjustment for several risk factors (BMI, smoking, history of diabetes, SBP, prevalent hypertension, CHD, or heart failure, alcohol consumption, and use of statins or calcium channel blockers) 1.00 (95% CI 0.59–1.69; *P* = 0.997). There was similarly no association after additional adjustment for SES and physical activity 1.00 (95% CI 0.59–1.69; *P* = 0.988) (Table 2). No significant associations were observed for diuretic use or β-blocker use with the risk of fractures. The association between ACEIs or ARBs use and composite fractures was not significantly modified by several clinically relevant characteristics (*P* for interaction ≥0.10 for each; Fig. 1). The corresponding adjusted HRs for hip fractures comparing ACEIs or ARBs use versus no use were 0.66 (95% CI 0.28–1.55; *P* = 0.338), 0.89 (95% CI 0.32–2.47; *P* = 0.820), and 0.89 (95% CI 0.32–2.47; *P* = 0.819) respectively. There was also no evidence of any associations with risk of wrist fractures (Table 2).

**Table 2:** Associations of use of ACEI or ARB and other antihypertensives with risk of fractures

	Events/total	Model 1		Model 2		Model 3	
		HR (95% CI)	*P* value	HR (95% CI)	*P* value	HR (95% CI)	*P* value
*Total fractures*							
No use	121/1093	ref		ref		ref	
Diuretic use	3/50	0.36 (0.11–1.14)	0.083	0.34 (0.10–1.10)	0.072	0.35 (0.11–1.13)	0.080
β-blocker use	52/351	1.11 (0.79–1.54)	0.547	1.06 (0.71–1.59)	0.771	1.07 (0.72–1.61)	0.729
ACEI or ARB use	27/249	1.00 (0.66–1.52)	0.992	1.00 (0.59–1.69)	0.997	1.00 (0.59–1.69)	0.988
*Hip fractures*							
No use	39/1093	ref		ref		ref	
Diuretic use	1/50	0.31 (0.04–2.28)	0.250	0.31 (0.04–2.36)	0.258	0.33 (0.04–2.50)	0.281
β-blockers use	24/351	1.39 (0.83–2.34)	0.209	1.76 (0.91–3.39)	0.093	1.81 (0.94–3.49)	0.078
ACEI or ARB use	6/249	0.66 (0.28–1.55)	0.338	0.89 (0.32–2.47)	0.820	0.89 (0.32–2.47)	0.819
*Wrist fractures*							
No use	30/1093	ref		ref		ref	
Diuretic use	1/50	0.51 (0.07–3.81)	0.513	0.72 (0.09–5.78)	0.755	0.73 (0.09–5.88)	0.769
β-blocker use	5/351	0.47 (0.18–1.22)	0.120	0.52 (0.18–1.54)	0.239	0.53 (0.18–1.57)	0.251
ACEI or ARB use	6/249	0.95 (0.39–2.28)	0.905	1.19 (0.39–3.64)	0.764	1.20 (0.39–3.68)	0.749

*ACEI* angiotensin-converting enzyme inhibitor, *ARB* angiotensin II receptor blocker, *CI* confidence interval, *HR* hazard ratio, *ref* reference

Model 1: Adjusted for age and sex

Model 2: Model 1 plus body mass index, smoking, history of diabetes, systolic blood pressure, prevalent hypertension, prevalent coronary heart disease, prevalent heart failure, alcohol consumption, statin use, and calcium channel blocker use

Model 3: Model 2 plus socioeconomic status and physical activity

#### Meta-analysis of published cohort studies

**Fig. 2:**
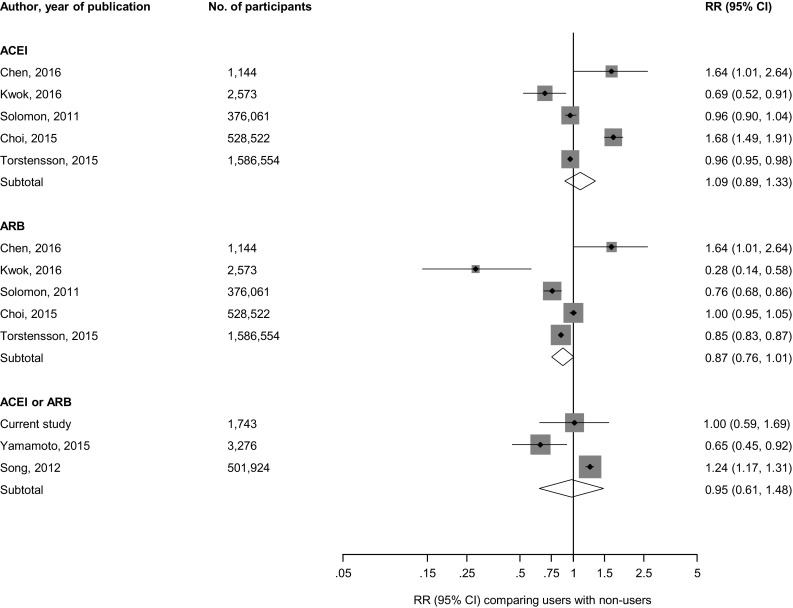
Prospective studies of RAS inhibitors and risk of composite fractures. The summary estimates presented were calculated using random effects models; size of data markers are proportional to the inverse of the variance of the relative ratio; *ACEI* angiotensin-converting enzyme inhibitor, *ARB* angiotensin II receptor blocker, *CI* confidence interval (*bars*), *RR* relative risk, *RAS* renin-angiotensin system blockers

Ten articles based on 10 unique cohorts were identified to have reported on the associations of ACEIs and/or ARBs and risk of fractures (Appendix 6 of Electronic Supplementary Material and Table 3) [22, 28, 29, 44–50]. Including the current study, there were 11 studies involving 3526,319 participants and >323,355 fractures. Quality scores of included studies ranged from 5 to 8. In pooled analyses of five studies each, the RRs for composite fractures comparing ACEI users with non-users and ARB users with non-users were 1.09 (95% CI 0.89–1.33) and 0.87 (95% CI 0.76–1.01) respectively. Comparing ACEI or ARB users with non-users, the RR for composite fractures in pooled analysis of three studies was 0.95 (95% CI 0.61–1.48) (Fig. 2). There was evidence of substantial heterogeneity (>75%) among the included studies in all pooled analyses.

**Table 3:** Characteristics of prospective studies included in meta-analysis

References	Name of study/source of participants	Location of study	Year(s) of baseline survey	Baseline age range (years)	% male	Mean/median duration of follow-up (years)	Total no. of participants	Fracture types	No. of fracture cases	Covariates adjusted for	Study quality
Solomon et al. [44]	Medicare beneficiaries	USA	NR	≥65	22.9	1.0	376,061	Composite, hip, wrist, humerus, and pelvic	6418	Age, gender, race, Charlson comorbidity score, number of physician visits, acute-care hospitalizations, number of different medications, osteoporosis diagnoses and medications, prior fractures, BMD testing, use of medications with fracture associations (e.g., oral steroids, anticonvulsants, benzodiazepines, selective serotonin reuptake inhibitors, and proton pump inhibitors), diagnoses associated with falls (e.g., Parkinson disease and Alzheimer disease), and prior history of falls	6
Song et al. [45]	KHIRAS database	Korea	2005–2006	≥65	35.0	1.0	501,924	Composite, hip, and vertebral	NR	Age, confounding comorbidities (osteoporosis, diabetes mellitus, hyperthyroidism, Cushing’s syndrome, COPD, asthma, chronic liver disease, chronic renal failure, congestive heart failure, dementia, stroke), and confounding medications (warfarin, antidepressants, benzodiazepines, anti-Parkinson drug, thiazolidinediones, bisphosphonates)	5
Thorell et al. [46]	CDWO	Sweden	2006	≥75	39.0	1.0	38,407	Hip	795	Age, gender, and multimorbidity level	5
Choi et al. [29]	HIRAS database	South Korea	2007–2011	≥50	48.6	1.9	528,522	Composite, vertebral, and non-vertebral	16,805	Age, gender, comorbidity score, diabetes, osteoporosis, osteoporosis treatment, and osteoporosis related diseases	6
Ruths et al. [47]	Norwegian Prescription Database; Norwegian Hip Fracture Registry; Central Population Registry	Norway	2005–2010	72.8*	44.0	5.2	906,422	Hip	39,938	NR	6
Torstensson et al. [48]	Danish Nation-wide Register	Denmark	1999–2012	≥65	81.2	6.7	1586,554	Composite	255,936	Age, gender, calendar year, comorbidities and exposure to the other classes of CVD-drugs	7
Yamamoto et al. [22]	MBD-5D Study	Japan	2008–2011	63.0*	61.5	2.7	3276	Composite	178	Age, sex, duration of dialysis, causes of end-stage kidney disease, BMI; Kt/V; comorbidity of cardiovascular disease and/or DM; smoking; history of parathyroidectomy; prescriptions of anticoagulants, vitamin D receptor activators, and phosphate binders; and serum levels of albumin, calcium, phosphorus, intact parathyroid hormone, alkaline phosphatase, and blood hemoglobin, in addition to systolic and diastolic blood pressure and the use of antihypertensive drugs (β-blockers, CCB, diuretics, and others)	6
Corrao et al. [49]	NHS	Italy	2005–2009	70–90	NR	5.0	81,617	Hip	2153	Use of antidepressants, neuroleptics, hypoglycemic agents, statins, digoxin, benzodiazepines, anti-arrhythmics, and anti-epileptics; Charlson comorbidity index	6
Kwok et al. [50]	MrOS	USA	2000–2002	≥65	100.0	6.8	2573	Non-vertebral and hip or wrist	801	Age, tricyclic antidepressants, thiazide use, previous fracture, inability to complete a narrow walk trial, falls in previous year, depressed mood, hip BMD, DM, cardiac failure, hypertension, duration of use of loop diuretic, statin, beta blocker and ARB (or ACEI)	7
Chen et al. [28]	TBNHI	Taiwan	2002–2012	65–80	43.6	11.0	1144	Composite	128	Age, sex, comorbidities, concurrent medication	
Current study	KIHD	Finland	1998–2001	53–74	47.6	14.8	1743	Composite, hip, and wrist	203	Age, sex, body mass index, smoking, history of diabetes, systolic blood pressure, prevalent hypertension, prevalent coronary heart disease, prevalent heart failure, alcohol consumption, statin use, calcium channel blocker use, socioeconomic status, and physical activity	8

*ACEI* angiotensin-converting enzyme inhibitors, *ARB* angiotensin II receptor blockers, *BMD* bone mineral density, *BMI* body mass index, *CCB* calcium channel blocker, *CDWO* care data

Warehouse in Östergötland; *CHD* coronary heart disease, *COPD* chronic obstructive pulmonary disease, *CVD* cardiovascular disease, *DM* diabetes mellitus, *KIHD* Kuopio Ischemic Heart Disease, *KHIRAS* Korean Health Insurance Review and Assessment Service database, *MrOS* Osteoporotic Fractures in Men Study, *NHS* National Health Service, *NR* not reported, *TBNHI* Taiwan Bureau of National Health Insurance, *USA* United States of America

**Fig. 3:**
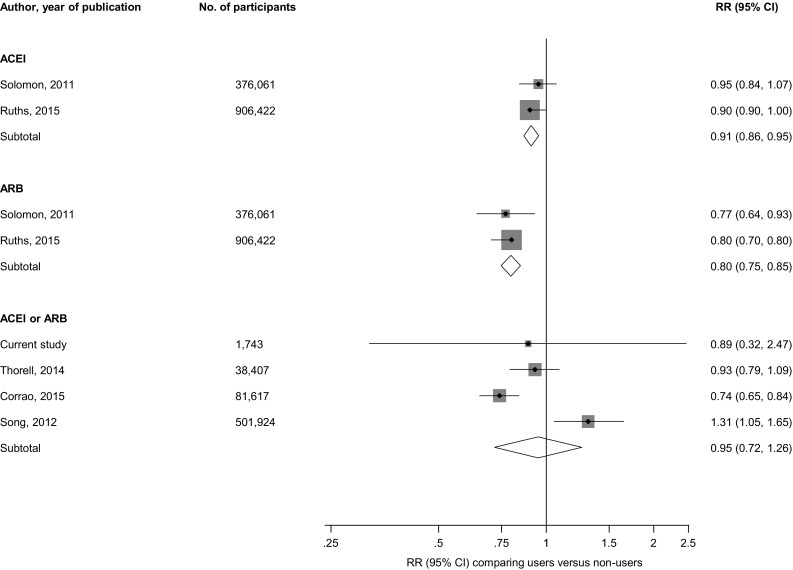
Prospective studies of RAS inhibitors and risk of hip fractures. The summary estimates presented were calculated using random effects models; size of data markers are proportional to the inverse of the variance of the relative ratio; *ACEI* angiotensin-converting enzyme inhibitor, *ARB* angiotensin II receptor blocker, *CI* confidence interval (*bars*), *RR* relative risk, *RAS* renin-angiotensin system blockers

The RR for hip fractures was 0.91 (95% CI 0.86–0.95) in pooled analysis of two studies (comprising 1,282,483 participants and 42,481 hip fractures) that compared ACEI users with non-users. In pooled analysis of these two studies, the corresponding risk was 0.80 (95% CI 0.75–0.85) when ARB use was compared with no use. Comparing ACEI or ARB users with non-users, the RR for hip fractures in pooled analysis of four studies was 0.95 (95% CI 0.72–1.26) (Fig. 3).

Comparing ACEI users with non-users, the RR for vertebral fractures was 1.69 (95% CI 1.40–2.04) and 0.81 (95% CI 0.69–0.95) for wrist fractures (Appendix 7 of Electronic Supplementary Material). The RR for wrist fractures was 0.68 (95% CI 0.53–0.87) and 0.76 (95% CI 0.61–0.94) for pelvic fractures, when ARB use was compared with non-use (Appendix 8 of Electronic Supplementary Material).

## Comment

### Summary of findings

In this population-based prospective study of middle-aged to elderly men and women, there was no evidence of an association of ACEI or ARB use with the risk of fractures and this was consistent across several clinically relevant subgroups. No evidence of significant associations with risk of fractures was also observed for diuretic use or β-blocker use. In pooled analysis of relevant published cohort studies, use of ACEI or ARB was not associated with the risk of composite fractures. However, pooled analysis of two large studies showed that ACEI use was associated with reduced risk of hip fractures [44, 47]. These two studies also showed that ARB use was associated with reduced risk of hip fractures. In pooled analysis of studies that specifically evaluated ACEI or ARB use, no evidence of decreased fracture risk was observed. The findings were inconsistent for other site-specific fractures; findings from some individual studies showed increased risk of vertebral fractures and decreased risk of wrist fractures with ACEI use [29, 44], whilst ARB use was associated with decreased risk of wrist and pelvic fractures [44].

### Comparison with previous work

Findings of our primary cohort analysis are consistent with a number of cohort studies [28, 44, 48] that have been published on the topic. There is however a possibility that our null findings for ACEI or ARB use as well as diuretic and β-blocker use could be due to the small sample that used these medications and the low event rates in these samples; therefore, the likelihood of insufficient power to demonstrate any potential associations. We are however unable to directly compare findings of our pooled analysis in the context of previous studies, as the current study is the first pooled analysis of published observational cohort studies evaluating the use of RAS inhibitors and the risk of fractures. In a recent pooled analysis of six case–control studies, Cheng and colleagues showed an increased risk of fractures with ACEI use and the association was stronger in older users (>65 years) [27]. However, given the case–control nature of the study designs, the temporal nature of the relationship is difficult to ascertain. The authors also called for cautious interpretation of the findings because of the substantial heterogeneity between the studies. Though our pooled analysis showed no evidence of an association of any of the RAS inhibitors with risk of total fractures; use of any of the RAS inhibitors was associated with reduced risk of hip fractures, but this was based on a limited number of studies. Therefore, further large-scale observational cohort studies are needed to confirm or refute the current findings.

### Possible explanations for findings

Given the close relationship between hypertension and its detrimental effects on bone physiology [6–10], it has been hypothesized that use of antihypertensive medications may be useful in preventing these fractures. Indeed, use of medications such as the thiazides and β-blockers have been consistently demonstrated to be associated with reduced risk of fractures [14–17]. However, findings from the primary cohort analysis showed no evidence of any association of diuretics or β-blockers with the risk of fractures, which could be attributed to the low event rates. Different mechanisms have been postulated for the protective effects of these medications on fracture risk. Thiazides modulate calcium homeostasis and lower urinary calcium excretion and therefore may reduce the risk of fractures via their effects on bone mass [51]. Evidence also suggests that thiazides have a direct effect on bone by promoting bone formation [52], whereas β-blockers may exert their beneficial effects on bone by blocking the adverse effects of the sympathetic nervous system on bone tissue [53, 54]. Emerging evidence also suggests that RAS inhibitors may reduce fracture risk, as the RAS has recently been shown to play a role in bone tissue. Components of the RAS such as angiotensin-converting enzyme (ACE) and angiotensin II are found locally in several tissues [55–57] and have been found to be expressed in osteoblasts and osteoclasts [58, 59]. This suggests the existence of a local RAS in the bone. Though angiotensin II has stimulatory effects on osteoblasts [60, 61], it has been generally suggested to have detrimental effects on bone structure by stimulating bone resorption [59]. Angiotensin II also decreases the uptake of calcium into bone [62], suppresses osteoblastic cell differentiation and bone formation [63], and decreases alkaline phosphatase activity [63]. Several studies in animal models have shown that inhibition of the angiotensin II signalling pathway may prevent osteoporosis [64], increase bone mass and strength [58, 65], and accelerate bone healing and remodelling [66].

Taken together, the evidence suggests ACEIs and ARBs, which inhibit the RAS, may help reduce fracture risk by improving bone composition and structure. The inconsistent associations between the use of RAS inhibitors and risk of site-specific fractures as demonstrated in our findings, may reflect the beneficial effects of angiotensin II on bone and the differential effects of ACEIs and ARBs on bone tissue. For example, Izu and colleagues showed in mice that a type 2 ARB significantly enhanced bone mass, whilst a type 1 ARB did not improve bone mass [58]. In the experimental study by Bayar and colleagues, ACEI was shown to have beneficial effects on fracture healing, whilst losartan, an ARB, failed to demonstrate comparable beneficial effects [67]. This differential effect may point to the differences in the function of both RAS-acting drugs. Further well-designed research is needed to delineate the mechanistic pathways by which these RAS inhibitors act on bone tissue.

### Strengths and limitations

Our study had the advantage of utilizing a large-scale population-based prospective cohort design with a pooled analysis of all published observational cohort studies on the topic in one comprehensive investigation. The primary cohort study employed a sample of men and women who were representative of the general middle-aged to elderly population; there was complete follow-up for all participants; follow-up period was long with annually updated incident outcomes; and the analysis was comprehensive with adjustment for several confounders as well as assessment for evidence of effect modification. Pooled analysis of previous studies, including the current study, enhanced power to assess the nature and magnitude of the association. Limitations of the current study include lack of separate data on ACEIs and ARBs and the duration of blood pressure treatment in the primary cohort; absence of data on relevant confounders such as markers of renal function (e.g., creatinine, estimated glomerular filtration rate), which influence bone health; the relatively young age of study cohort at baseline precluded the ability to potentially assess all fracture cases during the follow-up period of the current study, as majority of fracture cases tend to occur in the very elderly; the limited number of studies for the pooled analysis; inconsistent definition of composite fracture outcomes that did not enhance comparison across studies; substantial heterogeneity across studies; and inability to explore for publication bias because of the small number of studies. In addition, due to the variable adjustment by the eligible studies in the review, there was the possibility of residual confounding. However, the majority of studies reported estimates based on adjustment for a comprehensive panel of confounders. Finally, a number of these studies did not account for other antihypertensive medication use such as thiazides and β-blockers in their analysis.

## Conclusion

In a middle-aged to elderly population of Caucasian men and women, use of RAS inhibitors was not associated with risk of composite fractures and this was confirmed by our pooled analysis of previously published observational cohort studies. A beneficial effect of use of RAS inhibitors was observed for hip fractures, though the evidence was limited; however, it was based on pooled analysis of two large-scale cohort studies. Our study findings highlight the fact there are still inconsistencies in the associations of use of RAS inhibitors with risk of fractures. Further research is needed to confirm or refute these findings and assess the biological pathways underpinning any associations.

## Electronic supplementary material

Below is the link to the electronic supplementary material.

Supplementary material 1 (DOCX 158 kb)
